# Improvement of Biogas Production from Orange Peel Waste by Leaching of Limonene

**DOI:** 10.1155/2015/494182

**Published:** 2015-03-19

**Authors:** Rachma Wikandari, Huong Nguyen, Ria Millati, Claes Niklasson, Mohammad J. Taherzadeh

**Affiliations:** ^1^Swedish Centre for Resource Recovery, University of Borås, Allégatan 1, 50190 Borås, Sweden; ^2^Department Chemical and Biological Engineering, Chalmers University of Technology, 41296 Gothenburg, Sweden; ^3^Department of Food and Agricultural Product Technology, Faculty of Agricultural Technology, Universitas Gadjah Mada, Bulaksumur, Yogyakarta 55281, Indonesia

## Abstract

Limonene is present in orange peel wastes and is known as an antimicrobial agent, which impedes biogas production when digesting the peels. In this work, pretreatment of the peels to remove limonene under mild condition was proposed by leaching of limonene using hexane as solvent. The pretreatments were carried out with homogenized or chopped orange peel at 20–40°C with orange peel waste and hexane ratio (w/v) ranging from 1 : 2 to 1 : 12 for 10 to 300 min. The pretreated peels were then digested in batch reactors for 33 days. The highest biogas production was achieved by treating chopped orange peel waste and hexane ratio of 12 : 1 at 20°C for 10 min corresponding to more than threefold increase of biogas production from 0.061 to 0.217 m^3^ methane/kg VS. The solvent recovery was 90% using vacuum filtration and needs further separation using evaporation. The hexane residue in the peel had a negative impact on biogas production as shown by 28.6% reduction of methane and lower methane production of pretreated orange peel waste in semicontinuous digestion system compared to that of untreated peel.

## 1. Introduction

Orange as the main citrus fruit is one of top-five fruit commodities that dominate the global fruit market. According to Food and Agriculture Organization, global orange production reached 68 million tons representing 8.5% of the total fruit production [1]. The largest orange producers are Brazil, United States of America, China, India, and Mexico in 2012 [1]. Approximately, 40–60% of oranges are processed for juice production, of which 50–60% ends up as waste including seed, peel, and segment membrane [2, 3]. The generation of these solid wastes is estimated to be in the range of 15 to 25 million tons per year [3]. Among these wastes, citrus peel is the major constituent accounting for approximately 44% of the weight fruit mass [4].

Citrus waste for different applications such as production of pectin, flavonoid, fiber, and animal feed production has been proposed by several researchers [5–8]. However, a large amount of this waste is still dumped every year [9], which causes both economic and environmental problems such as high transportation cost, lack of dumping site, and accumulation of high organic content material [10]. Therefore, more effective and sustainable alternatives for using orange peel wastes such as biogas are highly desirable.

Biogas is gaseous material produced during anaerobic digestion of organic compound. Biogas holds wide applications such as fuel for electricity, car, cooking, lightening, and heating. Among these applications, conversion of orange peels wastes into fuel is attractive, since it gives benefits in terms of both energy recovery and environmental aspects. Orange peel waste contains both soluble and insoluble carbohydrates that can be digested to biogas [11]. However, the main challenge to produce biogas from orange peel is the presence of an antimicrobial compound “D-limonene.” This chemical constitutes 90% of oranges essential oil as 2-3% of dry matter of the orange [11]. Limonene has been reported to be highly toxic to anaerobic digestion [11–13]. It causes ultimate failure of the process at concentration of 400 *μ*L/L on mesophilic digestion [11] and in the range of 450 to 900 *μ*L/L on thermophilic digestion [14].

A number of investigations have been carried out to tackle the inhibition challenges by limonene [14, 15]. These methods can be classified into three categories of limonene removal, limonene recovery, and conversion of limonene into less toxic compound. Among these methods, limonene recovery seems to be the best alternative since this chemical is a valuable compound used in several industries such as perfumery, chemicals, cosmetics, medical, and food flavor [16, 17]. There are several methods that have been reported for limonene recovery including steam explosion [14], steam distillation [13], and acid hydrolysis [12]. However, these methods are performed under harsh conditions, which require high energy consumption. In addition, using acid for the pretreatment, a further neutralization is essential and expensive equipment should be applied to handle the corrosive behavior of the material. Furthermore, the acids used have a negative impact on the subsequent digestion process. On the other hand, since the goal of the pretreatment is to improve the biogas as a source of energy, the consumption of energy during the production process should be minimized. Hence, pretreatment of the orange wastes under ambient temperature is favorable.

Leaching or solid-liquid extraction is an alternative pretreatment performed in room temperature. In this technique, the limonene in the orange peel waste is leached into a solvent that has contact with the peel [18]. This technique is widely used to extract organic compounds from natural materials, where these compounds are present at low concentration. To the best of our knowledge, this technique has not been employed for pretreatment of orange peel wastes. Therefore, the objective of this work was to examine leaching technique for orange peel waste pretreatment with focus on biogas production.

## 2. Material and Methods

### 2.1. Material

Orange peel wastes were collected from Brämhults Juice AB (Borås, Sweden). The wastes were from orange juice process and contained 21.3% total solid (TS). It was then chopped or homogenized prior to pretreatment process. Inoculum was collected from a thermophilic biogas plant (Borås Energy and Environment AB, Borås, Sweden). The inoculum was kept at 55°C for 3 days before the digestion process. Chemicals including hexane, diethyl ether, and sodium sulfate were purchased from Sigma-Aldrich.

### 2.2. Methods

Pretreatment of the wastes by leaching was conducted in Erlenmeyer flasks. Four different solvents including hexane, diethyl ether, dichloromethane, and ethyl acetate were used. These chemicals are toxic and/or inflammable and should be treated properly. In the experiment to select the solvent, each solvent was added to the orange peel with orange peel waste and hexane ratio of 1 : 4. The mixture of solvent and orange peel was shaken vigorously for 10 minutes followed by incubation for 20 minute at room temperature. In the optimization of pretreatment study, hexane was used as a solvent. Two levels of four parameters including temperature (20°C and 40°C), time (10 min and 300 min), orange peel wastes and hexane ratio (1 : 2 and 1 : 12), and the wastes size (homogenized or chopped) were selected. Forty grams of the wastes was dissolved with a certain amount of hexane in the flasks, followed by shaking vigorously for a determined period of time. After the settlement, the extracts were removed from residuals by vacuum filtration. The residual, pretreated orange waste was then washed three times with water in order to remove remaining hexane. Finally, the pretreated waste was digested to produce biogas.

Digestion processes were performed in batch and semicontinuous reactors. The determination of biogas potential of the orange peels in batch digestion was carried out according to a previous study [19]. In the experiment to select the solvent, the digestion was conducted with different concentration of volatile solids (VS) ranging from 0.5 to 2%. In the optimization study, two percent of VS of the untreated and pretreated peels were placed in 120 mL glass bottle. The total volume of the mixture was 30 mL including 20 mL of inoculum and the rest was orange peel and water. The reactors were then flushed with a mixed gas containing 80% of N_2_ and 20% of CO_2_ for 2 min. The reactors were incubated at 55°C for 33 days. Reactors containing only water and inoculum were used as a blank. The experiments were performed in triplicate. At the end of the digestions, the pH of the digestates was measured. For semicontinuous digestions, the pretreated wastes were chosen based on the results obtained from the batch digestion. The semicontinuous digestions were performed in 2 L reactors (Automatic Methane Potential Test System I, Bioprocess Control, Sweden) with a liquid volume of 1.8 L. The reactors were placed in a water bath at 55°C. The organic loading rates (OLR) for both untreated and treated orange peels were set at 1 g VS/L/day during the starting up period and gradually increased to 3 g VS/L/day. The hydraulic retention time was set at 30 days. Gas production, pH, volatile fatty acids, and buffer capacity ratio were monitored during the digestion process.

Total solid and volatile solid of the untreated and pretreated wastes were determined using a gravimetric method. The gas production was measured using a gas chromatograph (Varian 450 GC, USA) equipped with a packed column (J&W Scientific GS-Gas Pro, 30 m × 0.320 mm) and a thermal conductivity detector (TCD). Gas samples of 100 *μ*L were withdrawn using a 250 *μ*L pressure tight syringe (VICI, Precision Sampling Inc., USA). The carrier gas was nitrogen with the flow rate of 2 mL/min. The temperature for injection, oven, and detector was 75, 100, and 120°C, respectively.

Hexane content of the orange wastes was analyzed by dissolving the wastes into 10 mL methanol. The methanol extract was then injected to gas chromatography-flame ionized detector (Clarus 400, Perkin Elmer) equipped with ZB-WAX-Plus, 30 m × 0.25 mm × 0.25 *μ*m.

For statistical analysis, normal probability method and analysis of variance (ANOVA) were performed using Design-Expert 8 package.

## 3. Results and Discussion

Orange waste is a potential feedstock for biogas production. Orange waste contains ca 74.5% carbohydrate, 7.7% protein, and 10.6% fat [20]. Even though the theoretical methane yield is 0.45 Nm^3^/kg VS, the methane yields of 0.061 and 0.131 Nm^3^/kg VS were obtained in this experiment from chopped and homogenized peel, respectively. This indicated the strong inhibition by the limonene. Therefore, this compound should be separated from the orange peel before the digestion process. In the current study, the limonene was recovered from the orange peel by solid-liquid pretreatment using solvent to extract the limonene.

### 3.1. Leaching of Orange Wastes and Subsequent Digestion

**Figure 1:**
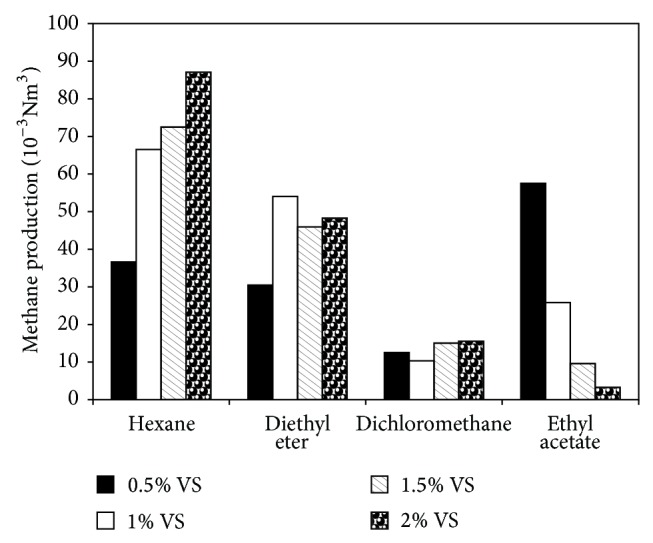
Methane production of pretreated orange peel waste by different solvents and digestion at different concentration of volatile solids.

In order to select a proper solvent for limonene recovery, four solvents including hexane, diethyl ether, dichloromethane, and ethyl acetate were used to extract the limonene followed by digestion of the pretreated orange peel waste for confirmation. The pretreated orange peel waste was digested at different concentration of volatile solids ranging from 0.5 to 2%. The results show that, for all VS concentration added, the orange peel pretreated with hexane gave the highest methane yield (Figure 1). Hence, the pretreatment using hexane was further investigated in order to obtain the best pretreatment method.

In the optimization study for pretreatment using hexane, four factors of leaching with two levels including temperature (20 and 40°C), time (10 and 300 min), orange peel waste and hexane ratio (1 : 2 and 1 : 12), and the citrus waste size (homogenized and chopped) were investigated. The pretreated orange waste was then digested to select the best condition of pretreatment. The results are summarized in Table 1. According to the statistical analysis, waste size was the only factor that was significant for the methane yield.

Table 1 showed that, for chopped peel, the pretreated wastes had higher methane production than the untreated ones. The pretreatment of the wastes increased methane production to the value of 0.076–0.217 m^3^/kg VS corresponding to 25–350% of improvement. The best pretreatment condition based on the methane yield obtained was for chopped peel treated at 20°C for 10 min with orange peel waste and hexane ratio of 1 : 12. This pretreatment increased the methane yield by more than three times. On the other hand, in the case of homogenized peel, the pretreatment resulted in lower methane production.

### 3.2. Hexane Inhibition in Digestion

**Figure 2:**
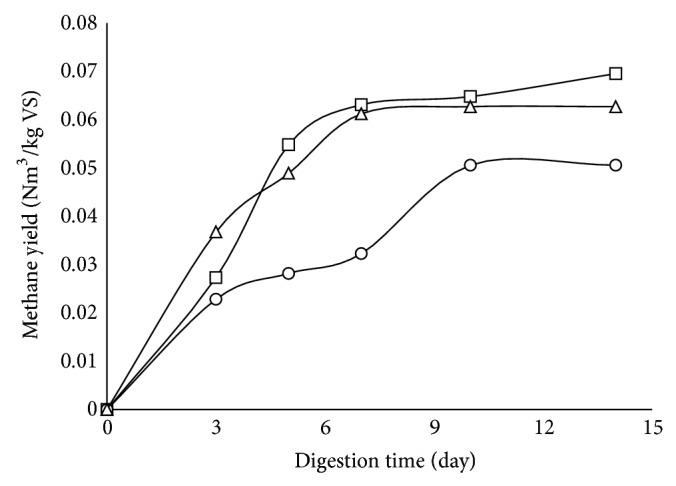
Effect of hexane (○) and limonene (Δ) on biogas production compared to control (□).

The toxic effect of hexane on anaerobic digesting microorganism might be responsible for the low yield obtained from the pretreated wastes. In order to confirm this hypothesis, batch digestion with addition of hexane to the digesting system was conducted. For comparison, the toxicity of limonene was also examined using the same method. The result showed that, at the same concentration, hexane was more toxic than limonene to anaerobic digesting system. Addition of hexane at concentration of 13 g/L resulted in 28.6% reduction of methane production compared with the control experiment (Figure 2).

The toxicity of hexane might explain the lower methane yield of the orange peel pretreated with homogenization. The smaller size of homogenized peels enabled greater contact surface between hexane and the peel resulting in higher hexane residue left in the peel. In addition, proportions of methane in biogas from pretreated homogenized orange wastes (45% to 68.5%) were lower than that of the pretreated chopped wastes (62.3% to 78.4%) (data not shown).

**Figure 3:**
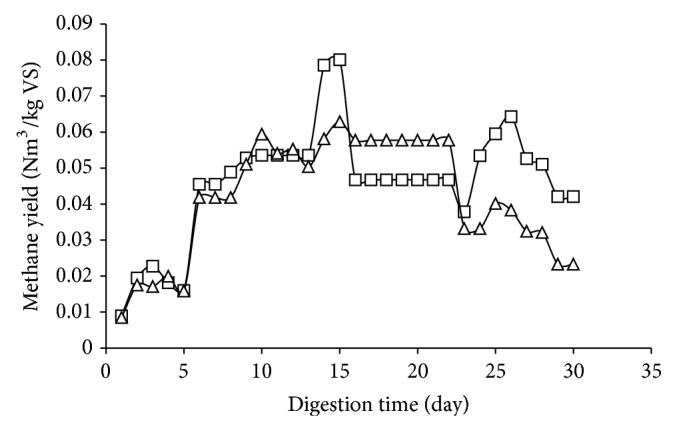
Methane production of untreated (□) and pretreated (Δ) orange peel wastes in semicontinuous digestion at organic loading rate of 3 g VS/L/day.

In order to further examine the accumulation effect of hexane in the system, semicontinuous digestion was conducted. The pretreated orange peel was compared with the untreated orange peel at organic loading rate of 3 g VS/L/day. Biogas production of the untreated and pretreated orange wastes is presented in Figure 3. The results show that the biogas production of the pretreated peel was lower than that of the untreated peel which might be due to the accumulation of hexane in the system.

### 3.3. Hexane Removal from Pretreated Orange Wastes

It was shown that hexane has inhibitory effect on anaerobic digesting system (Figure 2), and thus hexane residue in the peel must be removed prior to the digestion. Vacuum filtration was able to separate 90% of hexane and caused the hexane content of the pretreated orange wastes to be 0.2 mL/g of orange peel waste, which corresponds to concentration of 26 g/L hexane in the digesting system. This hexane residue was two times higher than the hexane concentration used in toxicity test (13 g/L). Hence, the hexane residue in the peel should be minimized or eliminated prior to the digestion process for both economic and technical reasons. Since hexane is a highly volatile hydrocarbon, removal of hexane can be performed by normal or vacuum evaporation process. In order to find the best condition, the evaporation was conducted at different temperatures and time. The range of temperature was between 30 and 70°C. Evaporation at temperature beyond 70°C made the orange peel very dried. In addition, boiling point of hexane is 68°C which is already below the maximum evaporation temperature. The low range of temperature gave advantages in which destruction of nutrients can be minimized and the energy consumption can be kept low. Hexane residue in the peel after evaporation was then analyzed. The result shows that 66% of hexane can be removed by evaporation at 50°C for 10 min corresponding to 9 g/L of hexane in the peel (Table 2).

### 3.4. The Overall Process of Methane Production and Limonene Extraction

One benefit of leaching pretreatment method is to recover the limonene that is a flavor compound in orange belonging to terpenoid group. As flavor compound, limonene holds widespread application in food, feed, cosmetic, chemical, and pharmaceutical industry. In the market of flavor, food and beverages is the largest which contributes to 47% of total demand in 2003 [21].

**Figure 4:**
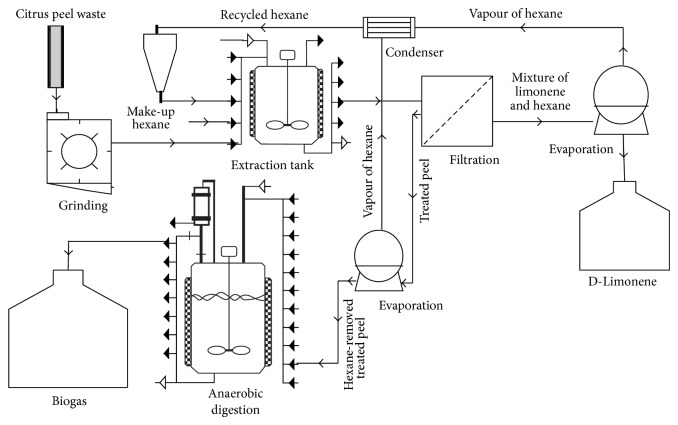
Block flow diagram of biogas production from treated orange peel waste by leaching pretreatment and limonene extraction.

In this process (Figure 4), orange peel is fed to grinding unit using a conveyer for size reduction. The chopped peel is mixed with hexane for 10 min at 20°C with peel and solvent ratio of 1 : 2, where limonene is extracted from the peels and dissolved in the organic phase of hexane. The peel is then separated from the hexane by vacuum filtration which separates ca 90% of the hexane. Since the remaining hexane in the peel inhibits the digestion, it should be separated and recycled using normal or vacuum evaporation. The treated peel is fed into anaerobic digester to produce methane. The mixture of hexane and limonene out from filtration which has about 0.55 L limonene per m^3^ of hexane is fed into rotary vacuum evaporator operated at 70°C in order to evaporate the volatile hexane and separate it from the limonene. The vapor of hexane is condensed and recycled back to the pretreatment vessel for extraction of more limonene from fresh peels.

The VS content of the treated orange peel is 11% and the methane yield was 0.177 Nm^3^/kg VS. Thus, every ton of orange wastes produced 19.47 Nm^3^ of biogas and 1.4 L of limonene as by-product. The best pretreatment condition obtained in this work increased the methane yield by 350% compared to the untreated peel. This improvement is lower than that obtained from steam-explosion pretreatment which could increase the methane yield by 426% [14]. However, in this current work the pretreatment was conducted at room temperature for 10 minutes, whereas the steam explosion pretreatment was carried out at 150°C for 20 minutes [14]. Thus, this pretreatment can be considered as low energy demanding. However, selection of the solvent is a critical point to avoid inhibition problem from the solvent on anaerobic digesting system.

## 4. Conclusion

Leaching of limonene from orange wastes can be a low energy demanding method for removing the inhibition effects of the wastes in the digestion process. The highest methane yield was obtained by pretreatment of the substrate at 20°C for 10 min with orange peel waste and hexane ratio of 1 : 12 which results in three times higher methane yield compared to the untreated wastes.

## Figures and Tables

**Table 1:** Methane yield of pretreated orange peel waste at different temperature, time, and peel/solvent ratio.

Temperature (°C)	Time (min)	Peel/solvent ratio	Citrus waste size	Methane yield (Nm^3^/kg VS)
Untreated			Homogenized	0.131 ± 0.008
20	10	1 : 2	Homogenized	0.101 ± 0.011
40	10	1 : 2	Homogenized	0.097 ± 0.009
20	300	1 : 2	Homogenized	0.040 ± 0.004
40	300	1 : 2	Homogenized	0.051 ± 0.010
20	10	1 : 12	Homogenized	0.071 ± 0.006
40	10	1 : 12	Homogenized	0.074 ± 0.006
20	300	1 : 12	Homogenized	0.094 ± 0.016
40	300	1 : 12	Homogenized	0.060 ± 0.014
Untreated			Chopped	0.061 ± 0.004
20	10	1 : 2	Chopped	0.177 ± 0.011
40	10	1 : 2	Chopped	0.162 ± 0.015
20	300	1 : 2	Chopped	0.134 ± 0.016
40	300	1 : 2	Chopped	0.102 ± 0.017
20	10	1 : 12	Chopped	0.217 ± 0.009
40	10	1 : 12	Chopped	0.076 ± 0.011
20	300	1 : 12	Chopped	0.120 ± 0.005
40	300	1 : 12	Chopped	0.121 ± 0.016

**Table 2:** Hexane residue in pretreated orange peel waste after evaporation in different conditions.

Pretreated peel waste	Evaporation temp. (°C)	Evaporation Time (min)	Hexane concentration (ml/g orange peel waste)	% hexane removal

Unevaporated (control)	—	—	0.12 ± 0.00	—
Evaporated	30	10	0.12 ± 0.01	0
Evaporated	30	30	0.06 ± 0.03	54
Evaporated	50	10	0.04 ± 0.00	66
Evaporated	50	30	0.07 ± 0.00	45
Evaporated	70	10	0.08 ± 0.02	31
Evaporated	70	30	0.09 ± 0.01	28
